# Machine learning for predicting the treatment effect of orthokeratology in children

**DOI:** 10.3389/fped.2022.1057863

**Published:** 2023-01-06

**Authors:** Jianxia Fang, Yuxi Zheng, Haochen Mou, Meipan Shi, Wangshu Yu, Chixin Du

**Affiliations:** ^1^ Department of Ophthalmology, The First Affiliated Hospital, Zhejiang University School of Medicine, Hangzhou, China; ^2^ Department of Orthopedic Surgery, The Second Affiliated Hospital, Zhejiang University School of Medicine, Hangzhou, China

**Keywords:** myopia, myopia control, orthokeratology, logistic regression model, nomogram, machine learning, artificial intelligence

## Abstract

**Purpose:**

Myopia treatment using orthokeratology (ortho-k) slows myopia progression. However, it is not equally effective in all patients. We aimed to predict the treatment effect of ortho-k using a machine-learning-assisted (ML) prediction model.

**Methods:**

Of the 119 patients who started ortho-k treatment between January 1, 2019, and January 1, 2022, 91 met the inclusion criteria and were included in the model. Ocular parameters and clinical characteristics were collected. A logistic regression model with least absolute shrinkage and selection operator regression was used to select factors associated with the treatment effect.

**Results:**

Age, baseline axial length, pupil diameter, lens wearing time, time spent outdoors, time spent on near work, white-to-white distance, anterior corneal flat keratometry, and posterior corneal astigmatism were selected in the model (aera under curve: 0.949). The decision curve analysis showed beneficial effects. The C-statistic of the predictive model was 0.821 (95% CI: 0.815, 0.827).

**Conclusion:**

Ocular parameters and clinical characteristics were used to predict the treatment effect of ortho-k. This ML-assisted model may assist ophthalmologists in making clinical decisions for patients, improving myopia control, and predicting the clinical effect of ortho-k treatment *via* a retrospective non-intervention trial.

## Introduction

Myopia has emerged as a major public health issue worldwide (1), with a higher prevalence in East Asia than in Europe and America. The condition may underlie several vision-threatening diseases such as cataract, glaucoma, retinal detachment, chorioretinal atrophy, and maculopathy, especially in high and pathological myopia (2, 3). Consequently, both optical and pharmacological treatments are vital for inhibiting axial elongation, thus decreasing potential complications. Orthokeratology (ortho-k) is popular in China due to its efficiency in controlling myopia in children, which is only second to muscarinic antagonists (4). However, the effect of ortho-k varies from child to child due to factors such as age, sex, axial length (AL), refractive error, ocular components, pupil diameters, and lens wearing time, among others (5). Although a few studies have reported the predictive quality of these baseline factors (5, 6), it is still unclear which factors are more accountable in estimating the long-term outcome of ortho-k treatments in children. A previous study has tried to predict the specific AL increase after wearing ortho-k, but there is no specific model to predict the efficacy of ortho-k (7).

This study, therefore, aimed to identify the characteristics and ocular components available to clinicians before the initiation of ortho-k. This information was used to construct a machine-learning (ML) -assisted (a subset of artificial intelligence) prediction model for the effects of ortho-k treatment.

## Methods

### Study design

This was a retrospective clinical trial conducted in The First Affiliated Hospital of the Medical College of Zhejiang University, based on patient records. Logistic regression prediction modeling, decision curve analysis, and receiver operating characteristic (ROC) were used to provide guidance for the evaluation of clinical treatment effects.

The study was conducted in conformance with the Declaration of Helsinki, and the study protocol was approved before initiation by the institutional review board of The First Affiliated Hospital of Medical College of Zhejiang University (IIT20220037B-R2).

### Participants

We collected the data of minor patients (*n* = 119) who received ortho-k treatment between January 1, 2019, and January 1, 2022, according to the study protocol. The exclusion criteria were: previous use of myopia treatments other than ortho-k; having a chronic disease, tumor, or injury after the treatment with ortho-k; and parents unwilling to take a telephone survey.

**Figure 1:**
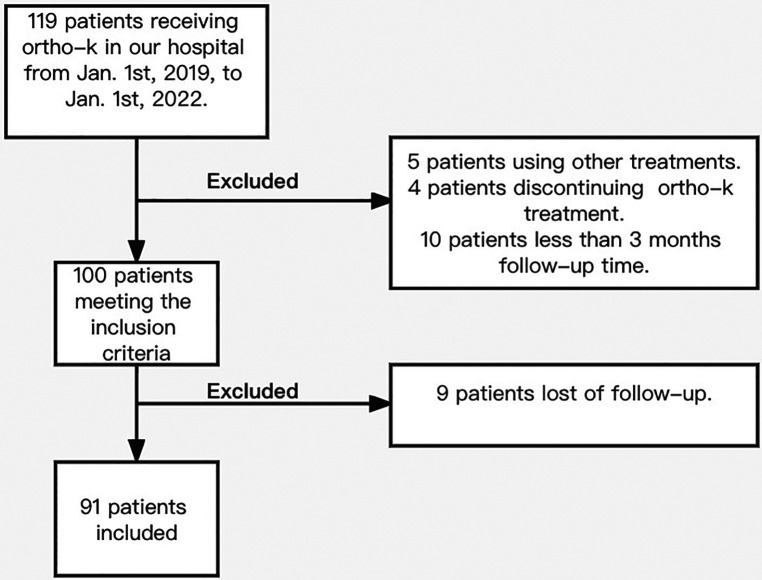
Flowchart of study.

Of the study population, five patients used other treatments, four patients discontinued ortho-k treatment, 10 patients were excluded due to follow-ups shorter than 3 months, and nine patients did not adhere to the follow-up (Figure 1). The minimum follow-up period among the remaining 91 patients who met the inclusion criteria was 3 months. Optometry and lens fitting were performed by the same doctor.

### Clinical data measurement

All clinical data were obtained with the high-resolution rotating Scheimpflug camera system for anterior segment analysis (Pentacam® HR, Oculus Optikgeräte GmbH, Wetzlar, Germany). Calibration frequency is every 2 years of use or every 25,000 times of uses, whichever is earlier. This analysis was conducted by the same physician in a dark room. The measurement was performed three times for each patient, and the average value was retrieved for analysis. AL measurements were performed by OA2000 (Tomey, Nagoya, Japan). Cycloplegia was then induced with 1–2 drops of Compound tropicamide (0.5% tropicamide plus 0.5% phenylephrine hydrochloride; Tianjin Kingyork Group Hebei Univision Pharmaceutical Co., Ltd., Hebei, China) instilled every 5 min over a 20-min period. Cycloplegic refraction was performed 45 min later by autorefraction (Nidek ARK-1, Nidek Co., Ltd., Aichi, Japan).

### Lenses

Three ortho-k brands were used in this study: Paragon corneal refractive therapy (CRT) (Paragon CRT, Paragon, Gilbert, AZ, United States); Dream Vision IV-DF (Dream Vision, Ovctek, Zhejiang, Anhui, China); and Alpha ORTHO-K (Alpha Orthokeratology, Alpha Corporation, Nagoya, Japan). CRT is made of paflufocon D (DK 100 × 10^−11^ [cm^2^/s]/[ml O_2_/ml × mmHg]) (ISO). Dream Vision is made of Boston XOP material with oxygen permeability of 100 × 10^−11^ (cm^2^/s)/(ml O_2_/ml × mmHg). Alpha ORTHO-K is made of Boston EM material with oxygen permeability of 104 × 10^−11^ (cm^2^/s)/(ml O_2_/ml × mmHg) (ISO). Lens fitting was performed according to the manufacturer's protocols based on corneal topography, cycloplegic refraction, and manifest refraction. Every patient wore trial lenses. They were observed under a slit lamp to ensure that the correct centration and refractive outcomes were achieved. The ordered lenses were dispensed to patients after 2–3 weeks, and patients were asked to return for follow up visits on the first day, first week, second week, first month, second month, and every 2–3 months thereafter. Every patient had corresponding complimentary lenses, solutions, and accessories to facilitate replacement in case of damage.

### Variables, outcome measures, information source

The myopia control effect of ortho-k was defined as the change in AL from the start of treatment. We defined a 1-year AL elongation ≤0.19 mm as the desired outcome, while an elongation >0.19 mm was defined as an unwanted outcome (1-year AL_elongation_ = AL_elongation_/months × 12) (8). The data of right eyes were included in our experiment. Ocular parameters, including spherical equivalent refraction (SER), baseline AL, white-to-white distance (WTW), pupil diameter, anterior chamber depth, anterior and posterior corneal steep and flat keratometry (flat K), anterior and posterior corneal astigmatism, anterior and posterior corneal high order aberrations (aberrations above 3rd order in Zernike polynomials) and central corneal thickness, were measured and used for our model. In addition, clinical characteristics, including age, sex, parental myopia history, time spent outdoors and near work every day, ortho-k brands, and lens-wearing time every night, were collected from patient medical records and telephonic surveys.

### Statistical analyses

Logistic least absolute shrinkage and selection operator (LASSO) regression was used to select factors related to the treatment effect. LASSO performs a continuous shrinking operation to minimize the regression coefficient and, consequently, the possibility of overfitting. By shrinking the sum of the absolute value of regression coefficients and compressing the coefficients to 0, this technique selects and retains the most relevant variables in the model (9). In our study, LASSO regression was performed for 100 cycles for all variables collected, and the variables retained more than 60 times were selected to determine the factors related to the treatment effect. The R package glmnet() function was utilized to fit the logistic LASSO regression.

The data were randomly divided in a training set-to-test set ratio of 7:3, thus establishing the logistic regression model. Secondly, the variables selected by LASSO were included in the nomogram. The discriminative ability was evaluated by the concordance index (C-index), which ranges from 0.5 (random chance) to 1.0 (perfect fit) (10). A calibration plot was used to evaluate calibrating ability: the prediction probability of the result is overestimated if the correction intercept is less than 0. On the contrary, if the correction intercept is positive, the algorithm is underestimated (11, 12). In addition, a decision curve analysis calculated the clinical net benefit for a given prediction model, comparing the default strategies for treating all or no patients (13). The “thin black line (none)” showed the expected net benefit where the intervention was not made, and the “thick black line (all)” showed the expected net benefit for all patients where the intervention was made (10). A heat map can intuitively display the relationship between variables and treatment effects.

K-fold cross-validation was carried out to evaluate the prediction ability of the model performances. To obtain the best estimate of model performance, a variant of the k-fold cross-validation technique was applied to the model, which would reduce the risk of overfitting. One-fold was used for validation, while the other k-1 folds were used to train the model and subsequently predict the target variables in the test data. Due to this process repeating k times (*k* = 10), the performance of each model in predicting the hold-out set was tracked using performance metrics such as accuracy, false-positive rate, and false-negative rate. The trainControl() function and train() function in the R package caret were used.

The ROC curve and confusion matrix were used to evaluate the performance of the model. SPSS software ver. 26.0 (IBM Corp., Armonk, NY, United States) and R software ver. 4.0.3 (The R Foundation Inc., Vienna, Austria) were used for the statistical analyses, curve drawing, and model building.

## Results

### Demographics

Table 1 shows the (median) demographic, ocular components, and clinical characteristics for all participants who met the study's final inclusion criteria (*n* = 91). The median post-treatment time was 12 months [interquartile range (IQR) 8–15 months].

**Table 1:** Demographic, ocular components, and follow-up clinical characteristics of included participants (*n* = 91).

Continuous variables	Median (interquartile range)	Categorical variables	Proportion
Age (years)	11 (9–13)	Sex (male: female)	46:45
Follow-up time (months)	12 (8–15)	Father's myopia history (high: moderate: low: no myopia)	19:25:29:18
SER at baseline (D)	−2.50 (−1.75 to −3.41)	Mother myopia history: (high: moderate: low: no myopia)	21:19:32:19
AL at baseline (mm)	24.66 (24.17–25.22)	Brands of Ortho-k (CRT: Alpha: Dream Vision)	46:35:10
Pupil diameter (mm)	3.50 (3.01–3.89)		
White-to-white (mm)	11.80 (11.50–12.00)		
ACD (mm)	3.25 (3.12–3.43)		
Time spent outdoors (hours)	1.3 (1.0–2.0)		
Lens wearing time (hours)	8.5 (8.0–9.0)		
Time spent on near work (hours)	8.5 (8.0–9.0)		
Anterior corneal steep K (D)	43.70 (42.80–44.50)		
Anterior corneal flat K (D)	42.40 (41.70–43.30)		
Anterior corneal astigmatism (D)	1.20 (0.90–1.50)		
Posterior corneal steep K (D)	−6.40 (−6.30 to −6.60)		
Posterior corneal flat K (D)	−6.10 (−6.00 to −6.30)		
Posterior corneal astigmatism (D)	0.30 (0.20–0.40)		
CCT (µm)	557 (533–577)		
Anterior corneal HOA (μ)	0.39 (0.33–0.46)		
Posterior corneal HOA (μ)	0.19 (0.18–0.21)		

SER, spherical equivalent refraction; AL, axial length; ACD, anterior chamber depth; K, keratometry; CCT, central corneal thickness; HOA, high order aberrations.

### LASSO regression for treatment effect

According to the result of feature selection by LASSO method, initial 22 features were reduced to 9 predictors, which retained more than 60 times. Therefore, the age, baseline AL, pupil diameter, lens wearing time, time spent outdoors, time spent on near work, WTW, anterior corneal flat K, and posterior corneal astigmatism were selected through LASSO. The coefficients of the variables filtered by LASSO showed its importance (Table 2).

**Table 2:** The LASSO regression selection for ortho-k treatment effect.

Variables	Coefficient
Age (years)	0.254
Baseline AL (mm)	0.068
Pupil diameter (mm)	0.146
Lens wearing time (hours)	0.834
Time spent on near work (hours)	−0.287

LASSO, least absolute shrinkage and selection operator; AL, axial length. Only the coefficients of variables that occur 100 times in 100 cycles are presented here. Other variables are omitted.

### Two-year logistic regression prediction model


$$p=\frac{1}{1+\exp\left(-A\right)}$$

The equation was derived from logistic regression, where *A* = [(−5.257 +1.577 age) − (0.419 baseline AL) + (1.557 pupil diameter) + (3.145 lens wearing time) + (1.542 time spent outdoors) − (2.928 time spent on near work) + (1.562 WTW) − (0.722 anterior corneal flat *K*) + (3.794 posterior corneal astigmatism)] (Table 3).

**Table 3:** Prediction model results using logistic regression.

Variables	Parameter			
	Estimate	Standard error	*Z* value	*Pr* (>|*z*|)
Age	1.577	0.534	2.952	0.003
Baseline AL	−0.419	0.901	−0.465	0.642
Pupil diameter	1.557	1.498	1.039	0.299
Wearing lens time	3.145	1.371	2.294	0.022
Time spent outdoors	1.542	1.437	1.072	0.284
Time spent on near work	−2.928	1.480	−1.979	0.048
WTW	1.562	2.343	0.667	0.505
Anterior corneal flat *K*	−0.722	0.672	−1.074	0.283
Posterior corneal astigmatism	3.794	4.819	0.787	0.431

AL, axial length; WTW, white-to-white distance; K, keratometry. The *Pr*(>|*z*|) value there corresponds to the *z*-statistic. The smaller the *Pr*(>|*z*|) value, the more significant the estimate.

**Figure 2:**
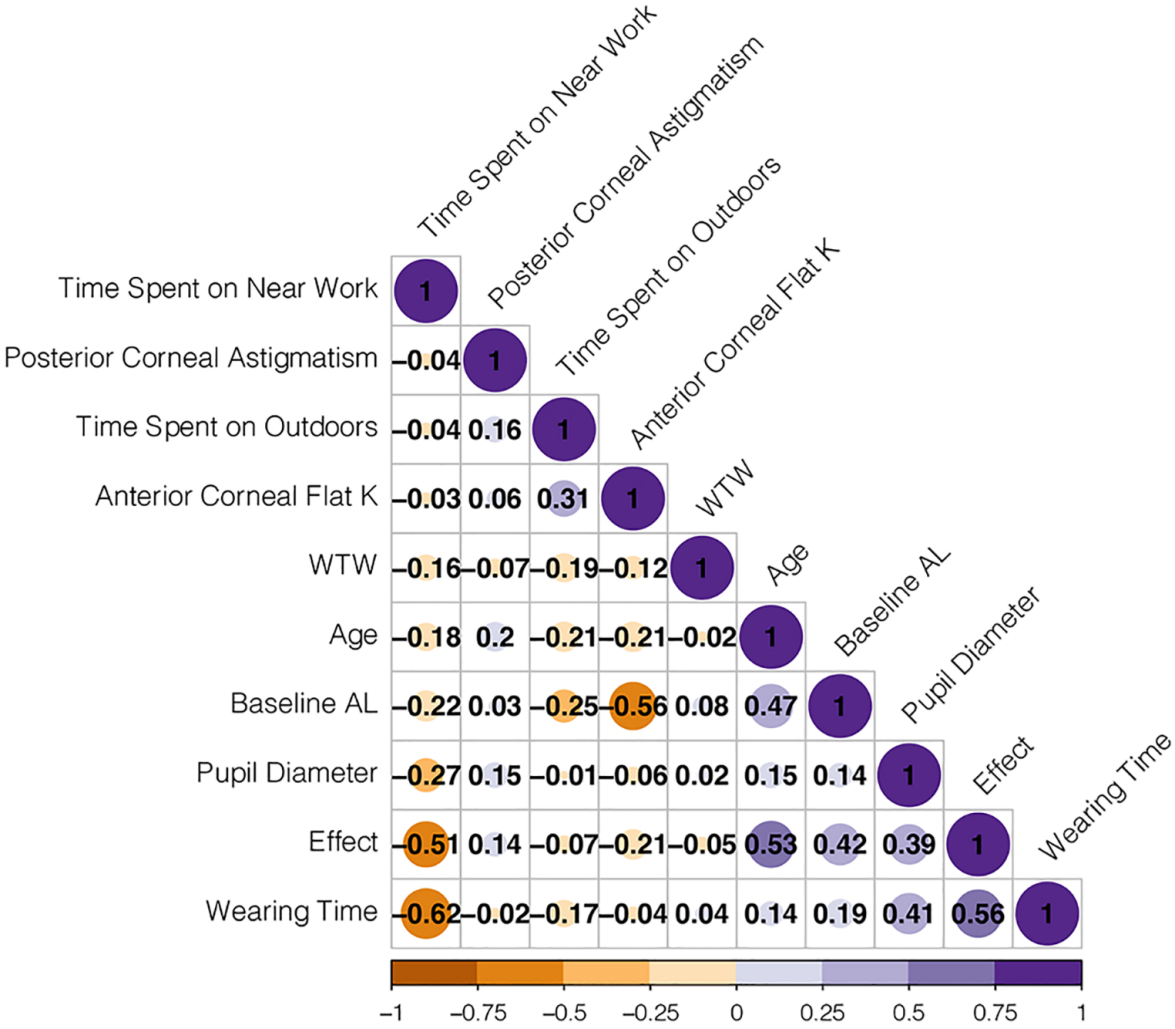
Heat map of correlation coefficient after logistic regression model. K, keratometry; WTW, white-to-white distance; AL, axial length. The figures are Pearson coefficients. The larger the absolute value of the figure, the stronger the relationship. The minus sign represents a negative relationship; the plus sign represents a positive relationship. For example, −0.51 represents a negative relationship between time on near work and effect.

**Figure 3:**
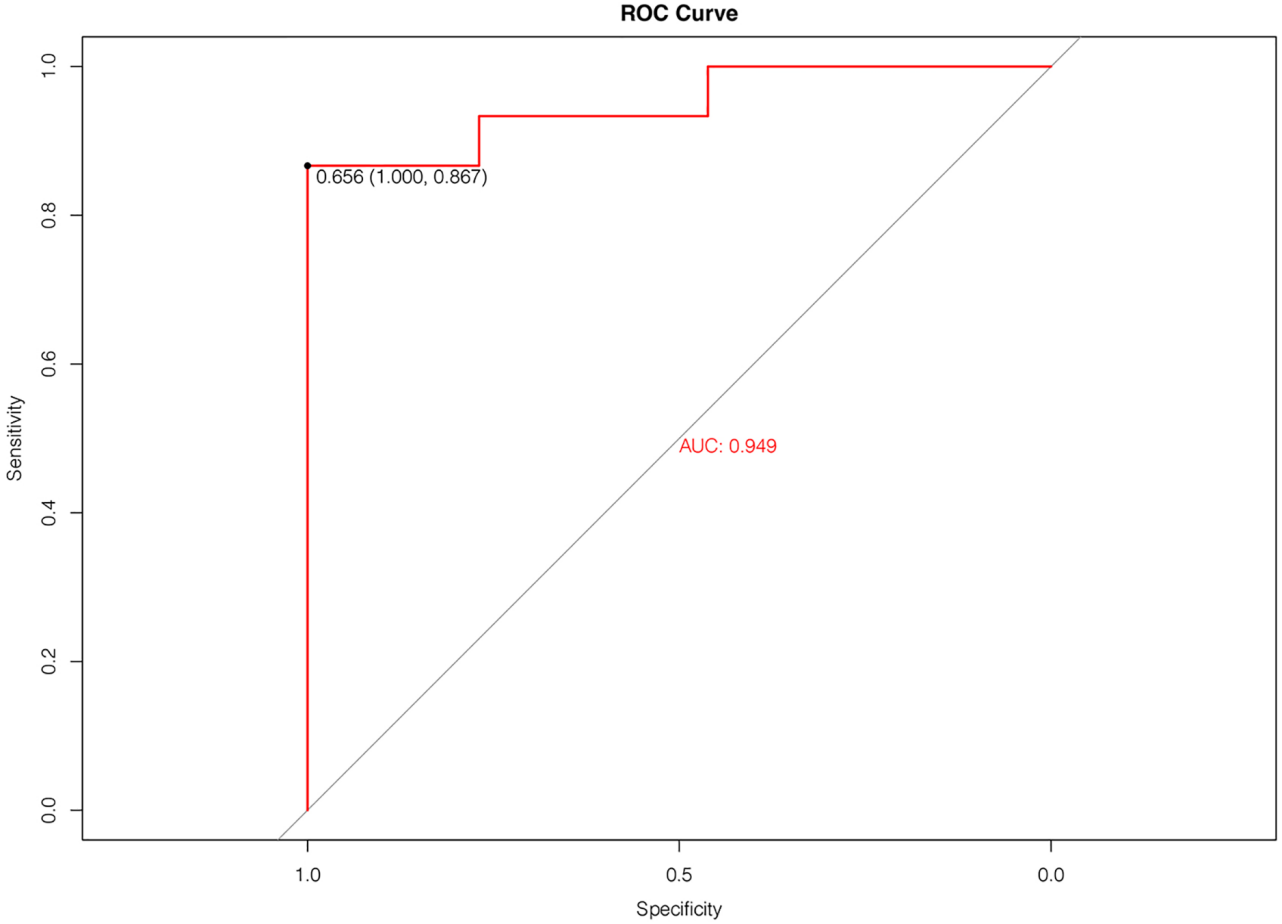
ROC curve of the prediction model we constructed.

**Figure 4:**
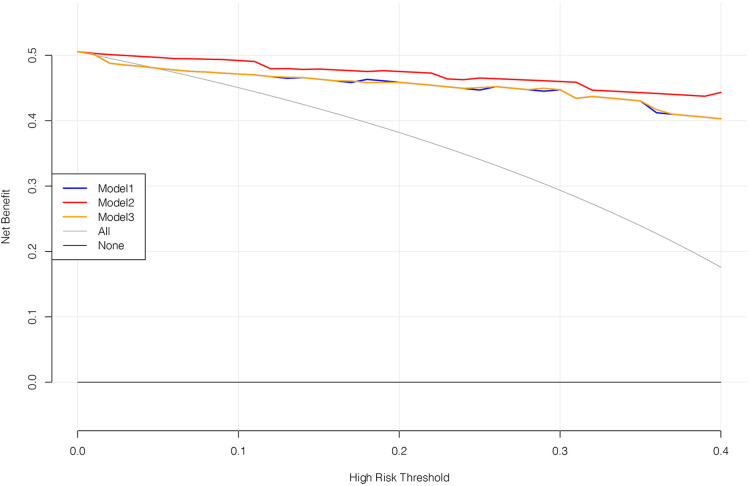
Decision curve analysis. Model 1 includes the age, baseline AL, pupil diameter, time spent outdoors, time spent on near work, and ortho-k brands. Model 2 is the model we constructed. Model 3 includes the age, baseline AL, pupil diameter, time spent outdoors, and time spent on near work. Among the three models, model 2 works best.

**Figure 5:**
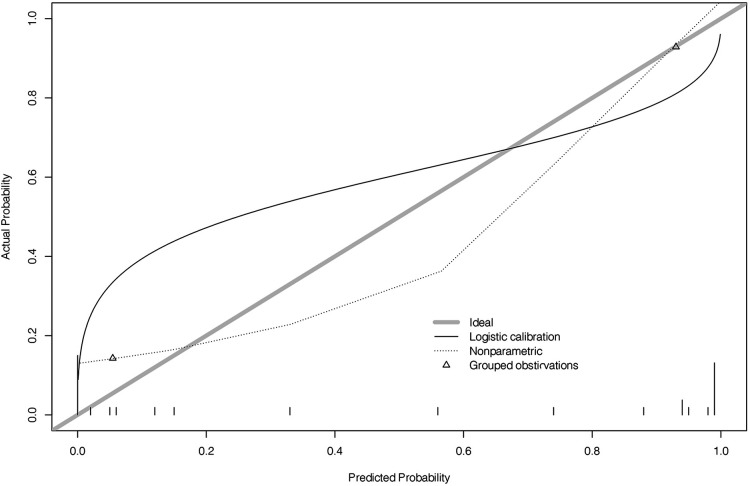
Prediction of cumulative effect. AL, axial length; WTW, white-to-white; K, keratometry.

**Figure 6:**
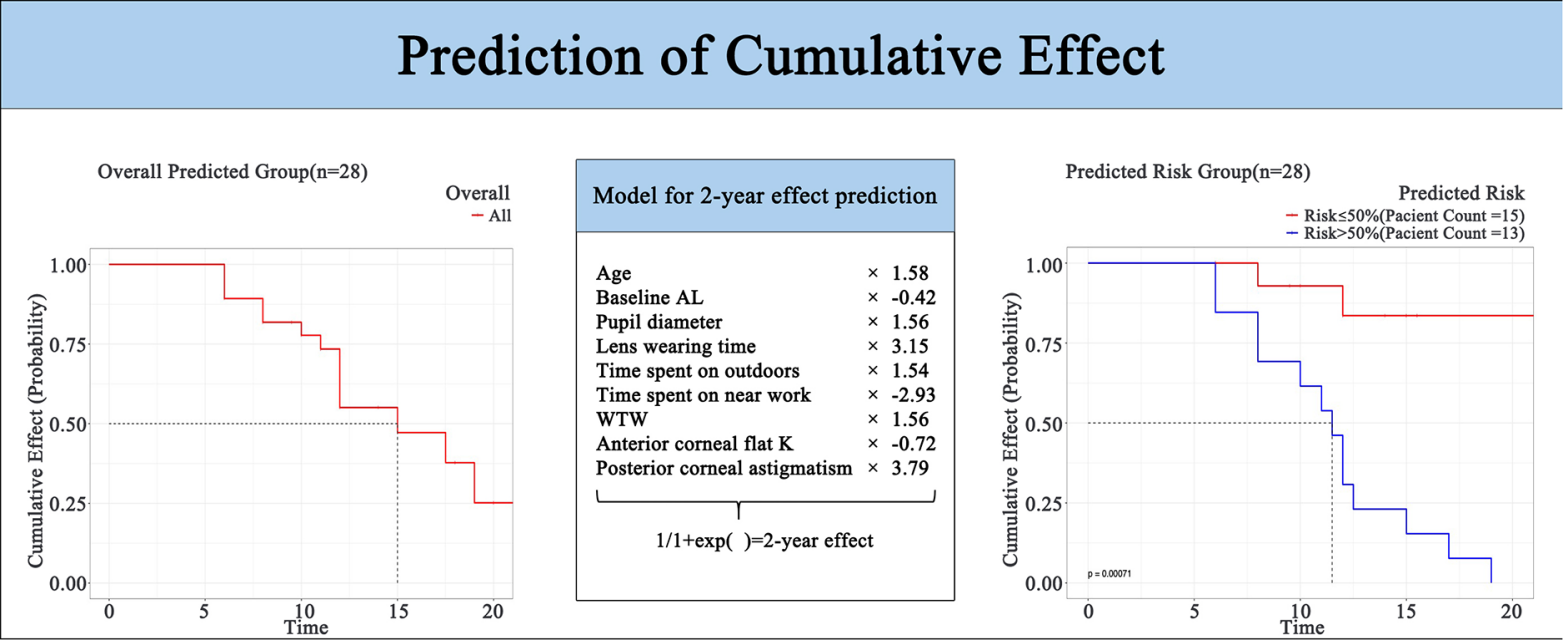
Nomogram of the 2-year possibility of the cumulative effect of ortho-k. K, keratometry; WTW, white-white distance; AL, axial length.

**Figure 7:**
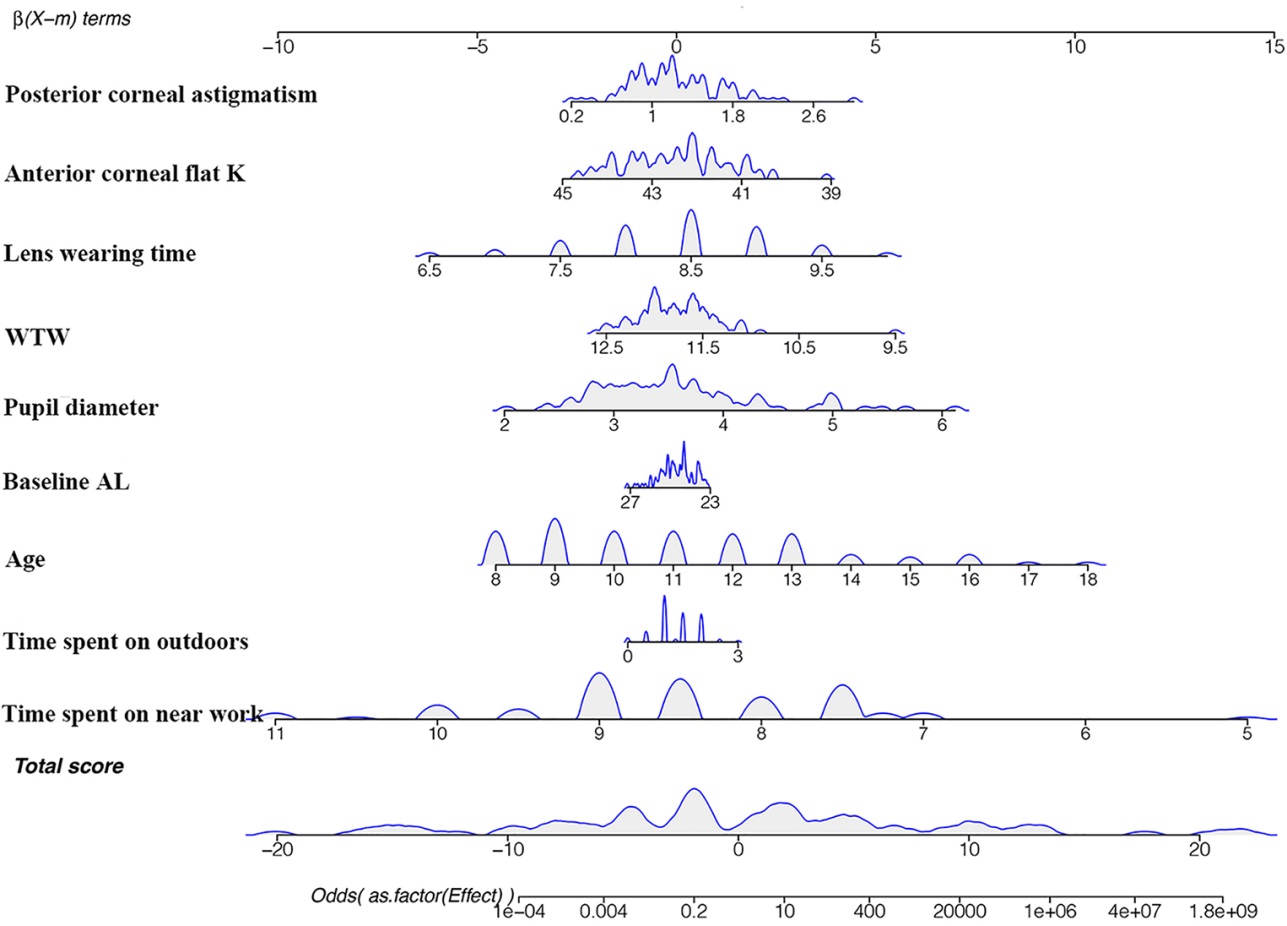
Calibration plot.

In the equation, $p=\frac{1}{1+\exp\left(-A\right)}$, *p* is the probability of cumulative effect. A cut-off value greater than 0.5 was effective. The estimates of each variable in logistic regression modeling prediction are shown in Table 4, showing the excellent predictive ability of the predictive model for good treatment effect (1). The heat map shows the correlation coefficients after the logistic regression model (Figure 2). Figure 3 shows the ROC, Figure 4 shows the decision curve analysis, and Figure 5 shows the calibration plot. Figure 6 presents the prediction of the cumulative effect. The 50% possibility of a cumulative effect was approximately 12 months. According to our model, we divided the patients in the validation set into two groups: risk ≤50% and risk >50%. The risk ≤50% group was more likely to obtain a better effect than the other group (*p* < 0.001). Figure 7 shows the nomogram for the possibility of the 2-year cumulative effect of ortho-k treatment.

**Table 4:** Logistic regression modeling prediction results.

	Prediction group		Correctly classified
Reference group	0	1	
0	13	2	86.7%
1	0	13	100%
Overall correct classified rate			93.4%

Accuracy: 0. 9286 (95% CI: 0.7650, 0.9912); Kappa: 0.8579; sensitivity: 0.8667; specificity: 1.0000. “0”, bad treatment effect, “1”, good treatment effect.

## Discussion

### Background and rationale

Although the mechanism underlying the myopia control effect is still unclear, peripheral myopia defocuses (14, 15) and the retardation effect of ortho-k has been widely recognized (15–17). Several possible factors have been previously associated with the effect of ortho-k (6), however, prediction models to estimate who will benefit from a better myopic retardation effect are lacking. Our prediction model quantified the myopia control effect classified by age, AL, pupil diameter, lens wearing time, time spent outdoors, time spent on near work, WTW, anterior corneal flat K, and posterior corneal astigmatism.

### Risk factors for cumulative effect

The variables, including clinical characteristics and ocular components, were screened as detailed in previous studies (5, 6). Kong et al. (6) reported that a smaller diopter and corneal eccentricity value were significantly associated with the treatment effect. Meanwhile, Santodomingo et al. (5) reported that, among other variables, earlier onset of myopia, female gender, lower initial myopia, a more prolate corneal shape, and larger pupil diameters constitute predictive factors for axial elongation in children wearing ortho-k. However, their conclusion was drawn by comparing the ortho-k group with a group wearing spectacles.

Interestingly, posterior corneal astigmatism was also associated with the ortho-k effect and ranked first in the model. Traditionally, ocular astigmatism, including posterior corneal astigmatism, has been considered for corneal refractive and cataract surgery due to its effect on postoperative visual function (18). Changes in posterior corneal astigmatism are associated with fluctuating changes in SER after corneal refractive surgery, but the effect is negligible for ortho-k treatments with minimal changes in corneal thickness and biomechanics. Of course, the long-term effects need to be further investigated.

Lens-wearing time ranked second in our model. The retardation of myopia progression improved when children's lens-wearing time increased, possibly because wearing ortho-k for longer leads to a more stable peripheral myopia defocus, resulting in a better myopia control effect.

Several studies had investigated the impact of environment and behavior management on myopia progression (19–22). Time spent on near work had the third greatest impact on the effect of ortho-k, and controlling it may significantly improve the treatment effect. The gradual extension of time spent on near work is a direct manifestation of the overloaded academic burden. In fact, people with higher education have a higher risk of myopia (23, 24). Time spent on near work was negatively associated with the cumulative effect of ortho-k. A meta-analysis by Lanca et al. (25) and Huang et al. (26) reports that time spent on near work and higher odds of myopia are positively correlated (odds ratio = 1.05 and 1.14), emphasizing the importance of managing this factor.

The impact of age on the effect of ortho-k ranked fourth in our prediction model. The nomogram (Figure 7) in our present study showed that the older the child, the better the effect. We speculated that as children age, the rate of myopia progression slows and generally stops at the age of 18. Consequently, the AL increase becomes smaller than at a younger age (27). This, however, does not mean that older children who begin wearing ortho-k will get better results than younger children. This result still suggests that the benefits of the ortho-k treatment increase if eligible children are subjected to it as early as possible.

The visible iris diameter often used in initial lens selection (28) was also associated with the ortho-k effect. Santodomingo et al. also confirmed that patients with larger iris diameters have better treatment effect with ortho-k (5). It is speculated that a larger iris diameter will prompt the prescription of a larger lens, which will form a larger defocus area, thus reducing more hyperopia defocus as described above. However, the specific mechanism still needs to be further studied in the future.

Pupil diameter has been positively associated with the cumulative effect of ortho-k (5, 29). Our results demonstrated that a larger pupil diameter with more significant redistribution of corneal epithelium from the corneal center to the peripheral cornea results in a larger reduction in peripheral hyperopic defocus (30, 31). Additionally, more defocused light tends to focus in front of the peripheral retina thus reducing axial elongation (14).

Increased time spent outdoors has been shown to protect against myopia onset and progression (19, 32, 33). The constantly increasing academic burden leaves school-aged children with less time for outdoor activities, sometimes limited to one physical education class (approximately 45 min). Our results indicated that the longer time invested in outdoor activities, the better the myopia control effect. Although there was little difference in the time spent outdoors of each child, it did have an impact on the therapeutic effect of ortho-k. This is similar to the result from a 3-year randomized controlled trial in Guangzhou, China, which suggested that more than 40 min of outdoor activities a day can reduce the incidence of myopia by 20% (19).

In clinical ortho-k practice, the corneal curvature, height, visible iris diameter, anterior corneal astigmatism, and eccentricity data retrieved from the corneal topography guide the first lens selection (28). In our nomogram, the smaller the anterior corneal flat *K* of the children, the better the effect. This result seems to contradict previous studies (5, 6). Santodomingo et al. (5) took the *p*-value (cornea shape factor) from the central 7 mm range of the cornea, and the larger corneal area taken into consideration may have led to a more significant relationship between *p*-value and myopia control effects. As for Kong et al.'s paper, they took the flat meridian of the whole eyeball and the corneal eccentricity value as the variables different from ours to identify factors influencing the therapeutic outcome of ortho-k (6). Therefore, these two values also affect the consistency of the results. In the future, we should try to expand the corneal range, as well as develop models including more predictive variables to predict the treatment effect more fully.

AL and SER are two major indicators reflecting the refractive state. An AL increase can serve as a good indicator of myopia progression because SER cannot be independently measured when wearing ortho-k (34). In most cases, the AL increase is in correlation with the SER (35). In the nomogram, the closer a child's AL is to 23 mm, the better the treatment effect. Considering the correlation between AL and SER, our results are consistent with the findings published by Santodomingo et al. (5).

In our study, different brands of ortho-k (mainly different designs, CRT and VST) had no significant effect on efficacy. In the future, the impact of different designs on the efficacy of ortho-k still needs to be determined by further randomized clinical studies with larger sample size.

### A 2-year logistic regression prediction model

Nomograms have been widely used in other medical specialties to predict the survival rate of patients and disease recurrence (36, 37). To construct the prediction model for treatment effect, we selected patients whose follow-up time was longer than 3 months (*n* = 91). The variables selected from LASSO regression were chosen to maximize the predictive power and used as a basis for the nomogram. LASSO regression can be used to predict the benefits of ortho-k treatment and recommend optimal myopia treatments or management for different children.

In this model, if the prediction model predicts “1”, ortho-k will be the most optimal treatment. If “0” is predicted, other myopia treatments instead of ortho-k will be recommended for the children. The optimism-corrected C-statistic of the prediction model was 0.821 (95% CI: 0.815, 0.827). The decision analysis curve showed that our model was good enough to guide lens fitting work in clinics (Figure 4). The calibration plots showed excellent overall agreement between the prediction, while observation of 2-year outcomes presented a good correlation between prediction and actual observation (Figure 5).

### Limitations

Our study has a few limitations. First, the sample size was relatively small, and because this was the first model to predict the cumulative effect of ortho-k, data for external validation was lacking. Second, our study only showed a potential association between pre-treatment variables and the cumulative effect. We did not explore the specific mechanism behind the correlation coefficient of each variable, which requires a larger sample to accomplish. Third, our study did not consider the corneal eccentricity value or the corneal shape factor *p* value within 7 mm of the central cornea; these need to be investigated in more depth in future studies.

## Conclusion

In conclusion, ML was applicable to predict the effect of ortho-k. We were able to predict a 2-year possible cumulative effect of ortho-k through age, baseline AL, pupil diameter, lens wearing time, time spent outdoors, time spent on near work, WTW, anterior corneal flat *K*, and posterior corneal astigmatism. This ML-assisted prediction model may instruct doctors' clinical decision-making for patients to achieve the best treatment effect.

## Data Availability

The original contributions presented in the study are included in the article/Supplementary Material, further inquiries can be directed to the corresponding author.
